# Lead in Paint: Three Decades Later and Still a Hazard for African Children?

**DOI:** 10.1289/ehp.9575

**Published:** 2006-12-14

**Authors:** Angela Mathee, Halina Röllin, Jonathan Levin, Inakshi Naik

**Affiliations:** 1 South African Medical Research Council, Johannesburg, South Africa; 2 World Health Organization Collaborating Centre for Urban Health, Johannesburg, South Africa; 3 National Institute for Occupational Health, Johannesburg, South Africa

**Keywords:** children, environment, lead, paint, South Africa

## Abstract

**Background:**

Surveys undertaken in South Africa have shown that a large proportion of children are exposed to lead from a variety of sources.

**Objectives:**

The overall objective of this work was to examine, through a series of small-scale investigations, the role of lead-based paint in the blood lead distribution of South African children.

**Discussion:**

We suggest that the African public health community strengthen their efforts to prevent lead poisoning in African children through a holistic approach that includes the promulgation and enforcement of appropriate legislation as well as research to identify further sources of exposure to lead.

In a 2002–2003 South African Medical Research Council survey of blood lead levels and associated risk factors in 383 first-grade Johannesburg school children, lead concentrations (apart from one extreme observation of 44.4 μg/dL) were shown to range from 1.0 to 18.1 μg/dL. The mean and median blood lead levels were 9.1 and 8.9 μg/dL, respectively, the interquartile range was 6.7–11.3 μg/dL, and 35% of children had blood lead levels ≥10 μg/dL (Mathee A, Rollin H, Levin J, Naik I, unpublished data). Peeling paint in homes was identified as a risk factor for elevated blood lead levels in children, as was pica for paint. The subject with the highest blood lead concentration was a 7-year-old girl whose repeat blood lead test, 3 weeks after the initial sampling, showed an increase to 51.5 μg/dL. Home assessments and interviews with her parents revealed a long history of pica for paint in particular (there was evidence of extensive paint removal in all the rooms of the apartment in which she lived), and lead concentrations up to 46,000 μg/g were measured in paint samples collected from her home (compared to the reference value of 5,000 μg/g) (Mathee et al. 2003).

## Results

The case outlined above raised concerns among the researchers of the potential for children’s exposure to lead-based paint in South Africa. Consequently, a survey was conducted by the South African Medical Research Council of the lead concentrations in paint samples collected from dwellings located in randomly selected Johannesburg suburbs. Of 239 dwellings included in the survey, 20% had paint lead concentrations > 5,000 μg/g (the U.S. reference level). Paint with high lead levels was found in old as well as newly constructed dwellings (Montgomery and Mathee 2005).

Suspecting the ongoing use of lead in paint in South Africa, researchers from this study purchased paint samples directly from Johannesburg and Cape Town stores, for lead content analysis. Although no lead was found in water-based or white shades of enamel paint, alarmingly high lead concentrations (up to 189,000 μg/g) were measured in samples of pigmented enamel paints. In total, 83% of the samples of pigmented enamel paints were lead based. High lead concentrations were found in popular as well as lesser-known brands of enamel paint, and only 2 of 25 samples of lead-based paint displayed warnings of the high lead content. Similarly high lead concentrations (up to 145,000 μg/g) were found in paint removed from widely used children’s toys (such as building blocks) that were purchased from major toy, supermarket, and stationery chain stores as well as flea and craft markets. High lead levels were found in locally manufactured as well as imported toys. On presentation of evidence of the elevated lead concentrations in paint on children’s toys, the Ministry of Health in South Africa acted to initiate a process, still ongoing, of drafting legislation to limit the use of lead in paint in the country.

## Discussion

Although on a small scale, the series of surveys and investigations outlined here made apparent the ongoing use of lead in paint in South Africa, and highlighted the gap in public health legislation required to protect children against this serious, yet preventable, environmental health hazard. Given that warnings of the risk to children from lead in paint were first published more than a century ago (Gibson 1904) and that steps to protect children against exposure to lead in paint were taken more than three decades ago in Australia, the United States, and elsewhere, it seems incomprehensible that children in South Africa may have been unnecessarily exposed to lead in paint for all this time. Despite global awareness of the hazardous nature of lead, and in contravention of their own voluntary lead restriction agreement, paint manufacturers in South Africa appear to have continued to use lead in certain paints. A direct consequence has been that large numbers of South African homes (especially those of the already vulnerable poor, who show a preference for enamel paint because of its relatively low cost and durability), children’s toys, and playground and educational equipment may be coated with lead-based paint. Environmental exposure to lead poses risks of intellectual impairment, poor educational attainment, and lowered lifetime achievement (Bellinger et al. 1987; Canfield et al. 2003; Needleman and Gatsonis 1990) for current and future generations of children in the country.

The legislation now being drafted in South Africa to restrict the use of lead in paint is laudable, but regrettably, comes too late for the many children who have, and will in the future be, unnecessarily exposed to lead in their homes and schools. The possibility exists that paint manufacturers in other African countries may similarly be continuing the hazardous and unethical practice of producing lead-based paint, and/or exporting or importing these products, and in so doing placing large numbers of children in Africa at risk of lead exposure and poisoning. In this regard, efforts by the authors have failed to identify the widespread existence and enforcement in African countries of legislation to control the use of lead in paint. Given the preventable nature of the use of lead and the serious consequences for children’s health and educational attainment, there is a need for greater vigilance and a more proactive approach to lead hazard prevention within the African public health community, including improved surveillance and research to identify the full extent of sources and risk factors, as well as implementation of the most appropriate lead poisoning prevention mechanisms.
